# Functional and taxonomic classification of a greenhouse water drain metagenome

**DOI:** 10.1186/s40793-018-0326-y

**Published:** 2018-10-05

**Authors:** Gamaliel López-Leal, Fernanda Cornejo-Granados, Juan Manuel Hurtado-Ramírez, Alfredo Mendoza-Vargas, Adrian Ochoa-Leyva

**Affiliations:** 1 Departamento de Microbiología Molecular, Instituto de Biotecnología, Universidad Nacional Autónoma de México, Avenida Universidad 2001, Colonia Chamilpa, Cuernavaca, 62210 Morelos Mexico; 2 Instituto Nacional de Medicina Genómica, Secretaría de Salud, Periférico Sur No. 4809, Col. Arenal Tepepan, Delegación Tlalpan, 14610 Ciudad de México, Mexico

**Keywords:** Shotgun sequencing, Greenhouse, Metagenome, Environmental sample, Water drain

## Abstract

Microbiome sequencing has become the standard procedure in the study of new ecological and human-constructed niches. To our knowledge, this is the first report of a metagenome from the water of a greenhouse drain. We found that the greenhouse is not a diverse niche, mainly dominated by *Rhizobiales* and Rodobacterales. The analysis of the functions encoded in the metagenome showed enrichment of characteristic features of soil and root-associated bacteria such as ABC-transporters and hydrolase enzymes. Additionally, we found antibiotic resistances genes principally for spectinomycin, tetracycline, and aminoglycosides. This study aimed to identify the bacteria and functional gene composition of a greenhouse water drain sample and also provide a genomic resource to search novel proteins from a previously unexplored niche. All the metagenome proteins and their annotations are available to the scientific community via http://microbiomics.ibt.unam.mx/tools/metagreenhouse/.

## Introduction

All the environments in the world contain millions of microorganisms. However, most of them are uncultivable, difficulting their study under laboratory conditions using traditional culture techniques. In contrast, the rapid development of sequencing technologies and the lower of their associated costs has allowed exploring the microbial composition of almost any ecological niche using metagenomic approaches, ranging from human gut to hot springs [1–3]. In this regard, metagenomic approaches have been used to answer two central questions: (i) which microorganisms are present and (ii) what is their functional contribution [4]. Metagenomic has opened the opportunity to find new microbial phyla [5] and novel protein families in previously unexplored niches [6], due to uncultivable microorganisms from there. Thus, the metagenomic resource provides the capacity of bioprospecting on the discovery of novel enzymes for research or industrial applications [7]. According to this idea, some new challenges in functional metagenomics, phylogenomics, ecology, and biotechnology have emerged. There are numerous applications of metagenomic analysis, ranging from prevention of diseases to solve industrial problems [8]. In recent years, the scientific community has tried to identify the role that microbial communities have in several disciplines such as human health [9, 10], and industry [11–13]. Metagenomics also has been applied to explore the impact of microorganisms in human-constructed niches [11, 14].

A greenhouse is an ecological niche entirely human manipulated, with the continuous exposure to pesticides, fertilizers, antibiotics and different chemicals for research purposes. Thus, subjecting the microbial communities under selective pressures. These effects can be analyzed using the high throughput sequencing methods. This allowed us the possibility to design new strategies for monitoring the microbial evolution of the structure and dynamics in particular human-constructed niches such as a greenhouse, plus comparing it to similar conditions somewhere else and eventually trace back any emerging problem. To our knowledge, this is the first report of a shotgun metagenome from a water sample of a greenhouse drain. Our work aimed to determine the microbial and functional composition of the water from a greenhouse drain. Our results indicated that this environment has low bacterial diversity, mainly dominated by *Alphaproteobacteria*, which is composed of *Rhizobiales* and *Rhodobacterales* orders. Interestingly, we found several antibiotic resistance genes and a functional enrichment for de novo amino acid synthesis in the metagenome.

### Site information

The sampling site corresponds to the water of a greenhouse drain. The greenhouse is on the top of a building, located at the Institute of Biotechnology (IBt) of the National Autonomous University of Mexico (UNAM), in Cuernavaca City in México. The greenhouse is used for the cultivation of several plant species for research purposes.

## Metagenome sequencing information

### Metagenome project history

The collected sample was part of a pilot project to identify the novel bacterial composition of the water in the experimental greenhouse drain at the Institute of Biotechnology (IBt) of the National Autonomous University of Mexico (UNAM). We deposited the sequencing reads in the NCBI under the SRA accession number SRR5689218 and SRR5689219 and the Bioproject PRJNA390663. Additionally, the reads were uploaded to the MG-RAST server under the ids mgm4717011.3, mgm4717032.3, mgm4716707.3, mgm4716832.3, mgm4716680.3, mgm4716681.3, mgm4716833.3, mgm4717034.3. For more details see the study information in Table 1.

**Table 1:** Study information

Label	Greenhouse Drain-IBt
MG-RAST ID	mgm4717011.3, mgm4717032.3, mgm4716707.3, mgm4716832.3, mgm4716680.3, mgm4716681.3, mgm4716833.3, mgm4717034.3
SRA ID	SRR5689218 (Drain A) SRR5689219 (Drain B)
Study	NA
GOLD ID (sequencing project)	NA
GOLD ID (analysis project)	NA
NCBI BIOPROJECT	PRJNA390663
Relevance	Water drain sample

### Sample information

We collected the sample on 14 September 2015 at 18:00 h (GMT-5) at the IBt (Latitude: 18.918611, Longitude: − 99.234167). In Table 2 the sample information according to the minimal information standards is showed [15].

**Table 2:** Sample information

Label	Greenhouse Drain-IBt
GOLD ID (biosample)	NA
Biome	Culturing environment
Feature	Water of greenhouse drain system
Material	Water
Latitude and Longitude	18.918611, −99.234167
Vertical distance	1510 m over sea level
Geographic location	Cuernavaca, Morelos. México
Collection date and time	14/09/15, 18:00 h (GMT-5)

#### Sample preparation, DNA extraction, library generation, and sequencing technology

##### Sample preparation (collection, transport, and storage)

A sample of 170 ml of water was directly collected from the greenhouse drain and immediately transported to the laboratory, located in the same building. Microbes were obtained by filtering this water through a sterilized PTFE 0.45 μm filter (Cat. 728–2045, Nalgene, NY, USA) using a vacuum pump. After filtration, we extracted the total DNA from the membranes.

### DNA extraction (kits used, protocols used)

Total DNA was recovered from the filter membrane by shaking the filter for 5 min in a tube containing lysis solution and beads from ZR Soil Microbe DNA MicroPrep Kit (Cat. D6003 Zymo Research, Irvine, CA, USA). The following steps for DNA isolation were carried out following the manufacturer’s instructions for the ZR Soil Microbe DNA kit. After extraction, we assessed the DNA quality by agarose gel electrophoresis and quantity determined by the Thermo Fisher Qubit High-sensitivity fluorometric assay (Cat. Q32851, Life Technologies, Carlsbad, CA, USA).

### Library generation (kits used, protocols used)

We constructed two DNA libraries containing different insert sizes: Drain-A and Drain-B with an insert size of 400 and 2000 bp, respectively (Table 3). Furthermore, different amounts of input DNA were used to construct the libraries: 1 ng for Drain-A and 25 ng for Drain-B. Both libraries were created following the manufacturer’s instructions for the Nextera XT DNA Library Preparation kit (Cat. FC-131-1024, Illumina, CA, USA). First, DNA was fragmented (tagmented) using the Nextera transposase. Second, the tagmented DNA was amplified using 12 PCR cycles to add the Index 1 (i7), Index 2 (i5), and full adapter sequences. The program on the thermal cycler was as follows: 72 °C for 3 min, 95 °C for 30 s; 12 cycles (95 °C for 10 s, 55 °C for 30 s and 72 °C for 30 s) and 72 °C for 5 min. After PCR amplification, both libraries were carefully size selected using Agencourt Ampure XP beads (Cat. A63882, Beckman Coulter, CA, USA) and the size was verified using a DNA Agilent Bioanalyzer 2100 (Cat. 5067–1504, Agilent Technologies, CA, USA).

**Table 3:** Library information

Label	Drain-A	Drain-B
Sample Label(s)	Drain-A	Drain-B
Sample prep method	ZR Soil Microbe DNA (Zymo)	ZR Soil Microbe DNA (Zymo)
Library prep method(s)	Nextera XT	Nextera XT
Sequencing platform(s)	Illumina NextSeq 500	Illumina NextSeq 500
Sequencing chemistry	V2 SBS Kit	V2 SBS Kit
Sequence size (GBp)	10.4GBp	0.60GBp
Number of reads	6,976,736	401,466
Single-read or paired-end sequencing?	Paired-end	Paired-end
Sequencing library insert size	500 bp	2000 bp
Average read length	150 bp	150 bp

### Sequencing technology

The Illumina NextSeq 500 Mid Output cell was used for sequencing in a 2 × 150 bp paired-end format, resulting in a total of 7,378,202 of reads for a sum of 11 Gbp of DNA data. Each sample yielded 6,976,736 and 401,466 of reads for Drain-A and Drain-B libraries, respectively (Table 4).

**Table 4:** Sequence processing

Label	Greenhouse Drain-IBt (merged library name)
Tool(s) used for quality control	Fast QC, Dynamic Trimm
Number of sequences removed by quality control procedures	169,936
Number of sequences that passed quality control procedures	7,208,266
Number of artificial duplicate reads	664,856

## Sequence processing, annotation, and data analysis

### Sequence processing

Pair-end raw reads were quality filtered using DynamicTrimm [16]. To this end, we eliminated the barcodes and primers, removed the reads containing ambiguous bases and trimmed the sequences with quality >Q20 (6 bp sliding window). We mapped the raw reads against *Homo sapiens* genome (GRCh38) using BWA with default parameters [17] to remove human DNA for downstream analysis.

### Metagenome processing

All the quality-filtered reads of the two libraries were used to construct two de novo metagenomic assemblies, one using IDBA-UD [18] with 20–125 of *k-mer* length range and other using MetaSpades [19] with a k-mer range of 21–121 with steps of 10 (Table 5). After that, we used Mummer (nucmer) with a cluster match of 80 nucleotides (contig coverage) and 99% of identity to merge all the contigs of both assemblies [20]. The merged metagenome was selected because it contained a minor number of contigs and the best N50 and L50 among the others. The contigs were validated mapping back the reads using BWA [17] with default parameters, resulting 79% of the reads mapped back to the final assembly with coverage of 26X [21]. This percentage and coverage are adequate for a metagenome assembly [22]. All the contigs larger than 1 Kb were selected for gene prediction and functional annotation, resulting in 7003 contigs with an N50 and N75 of 4246 and 1807 bp, respectively.

**Table 5:** Metagenome statistics

Label	Metagenome Label	Comment
Libraries used	Drain-A and Drain-B	We performed the assembly using all the reads of the two libraries that passed quality filters.
Assembly tool(s) used	IDBA-UD and MetaSpades and merged with nucmer	20–125 of *k-mer* length (IDBA-UD) 21–121 (MetaSpades)
Number of contigs after assembly	7003	These numbers correspond to the best assembly merged using nucmer.
Number of singletons after assembly	N/A	MetaSpades and IDBA-UD were used in pre-correction mode to discard singletons k-mers.
Total bases assembled	859,091,400	Total base pairs in the assembly.
Contig n50	4246	
% of Sequences assembled	97%	The fraction of the input data in the assembly.
Measure for % assembled	79%	The method used for calculating % assembled was determinate by read mapping using BWA (default parameters) against final assembly and considering the total reads (7,208,266 reads)

### Metagenome annotation

All classified reads (at different taxonomic levels) for each library were merged into a single library (Greenhouse Drain-IBt) to determine their relative abundances. After that, all quality-filtered reads were functionally and taxonomically classified using the MG-RAST server [23]. The annotations are available under the accession numbers mgm4717011.3, mgm4717032.3, mgm4716707.3, mgm4716832.3, mgm4716680.3, mgm4716681.3, mgm4716833.3, mgm4717034.3. The taxonomic and functional classification was performed with  MG-RAST server using the RefSeq and SEED subsystem databases with default parameters, respectively (Table 6). Normalized raw count was used to determine the relative abundances of reads for each taxonomic level, using an in-house developed Perl script. Additionally, the reads were also taxonomically classified by Kraken [24] using the RefSeq bacterial database from NCBI (ftp://ftp.ncbi.nlm.nih.gov/genomes/refseq/bacteria/). Taxonomic abundances were calculated using an in-house developed Perl script based on the number of reads for each taxonomic group. Furthermore, the reads were also functionally annotated by HUMAnN2 using the UniRef90 database. The taxonomic association in HUMAnN2 [25] was performed with Meta PhlAn2 using ChocoPhlAn database.

**Table 6:** Annotation parameters

Label	Metagenome Label	Comment
Annotation system	Drain-IBt	The functional annotation using the reads was obtained using MG-RAST, Kraken, and HUMAnN2, while the functional protein annotation of the assembly was obtained from GO, InterPro, and KEGG using Blast2GO.
Gene calling program	Frag Gene Scan	FragGeneScan was training with Illumina reads.
Annotation algorithm		
Database(s) used	RefSeq, SEED, ChocoPhlAn, UniRef90, Interpro (data bases) Blast NR data base	

### Post-processing

Final contigs of the metagenome were used to predict 25,735 proteins by FragGeneScan (Table 7) [26]. Out of the total of predicted proteins, 21,700 were functionally annotated by Blast2GO PRO version 2.8 [27], using BLASTp against NR, Gene Ontology (GOs) and InterProScan version 5.25 [28]. Antibiotic resistance genes (ARGs) were determined using the antibiotic resistance genes database (ARDB).

**Table 7:** Metagenome properties

Label	Metagenome label	Comment
Number of contigs	7003	
GBp	11.0 GBp	
Number of features identified	25,735	Total number of predicted protein features from the assembly
CDS	21,700	Total number of proteins annotated by Blast2GO and Interpro.
rRNA	18,612	Total number of reads determined as ribosomal genes using RiboPicker version 0.4.3.
CDSs with GO	14,328	Number of proteins with GO terms. Number of reads mapped to a protein.
CDSs with UniRef90	1,619,062	
CDS with SEED subsystem	786,622	
Alpha diversity	2.04 and 1.99	Alpha diversity was determinate at order level comparing MG-RAST and Kraken results. Shannon index was measured using Phyloseq.

## Metagenome properties

Shotgun sequence data generated a total of 7,378,202 of reads that were quality processed (see Sequence processing section) and got 7,206,754 of quality reads. Next, we assigned the taxonomy classification to 1,966,261 reads using MG-RAST and to 877,380 reads using Kraken, both of them using the LCA method [29]. Additionally, high-quality reads were used to obtain a de novo assembly consisting of 7003 contigs with an N50 and N75 of 4246 and 1807 bp, respectively. The largest contig of the assembly had 221,208 bp length (Table 5).

### Taxonomic diversity

**Fig. 1:**
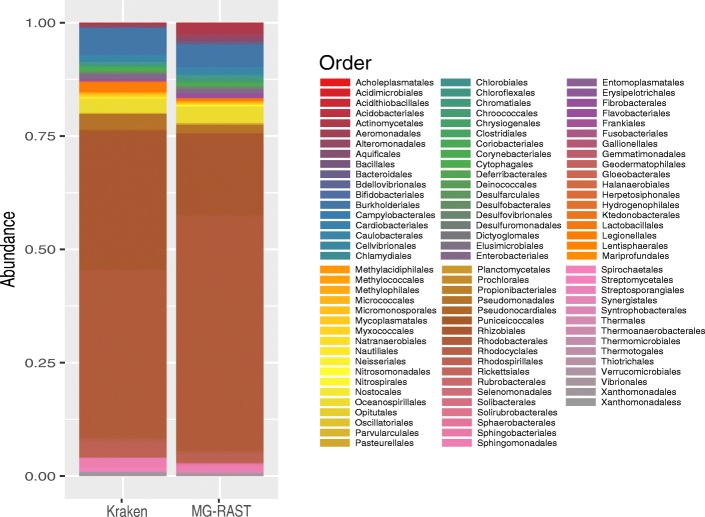
Bacterial abundance at order level. Relative abundance of bacterial orders classified by Kraken and MG-RAST

After using MG-RAST, we found that *Proteobacteria* was the most abundant phylum with 91% of the reads, followed by *Actinobacteria* (2.6%) and *Bacteroidetes* (2%) (Table 8). Additionally, at the class level, *Alphaproteobacteria* (74%) was highly present, and *Gammaproteobacteria* (10%), *Betaproteobacteria* (6%), and *Actinobacteria* (3%) showed the lowest abundances. The *Rhizobiales* and *Rhodobacterales* orders (*Alphaproteobacteria*) were the most abundant in the metagenome (Fig. 1). Next, we used Kraken to compare the taxonomy classification obtained by MG-RAST. Although Kraken assigned a lower number of reads (877,380) than MG-RAST (1,966,261), we found similar results in taxonomy abundance. *Proteobacteria* was the most abundant phylum (94%), followed by *Actinobacteria* (3%) and *Bacteroidetes* (0.4%) (Table 8). Also, similar to MG-RAST classification, *Rhodobacterales* and *Rhizobiales* orders were highly abundant. The most critical difference in taxonomic classification between the two algorithms was the number of orders identified, MG-RAST identified 92 and Kraken 51 orders (Fig. 1). To evaluate if this difference could impact on the microbial alpha diversity metrics, we used the relative abundance tables from MG-RAST and Kraken to measure the richness (Simpson) and evenness (Shannon) using Phyloseq [30]. This analysis showed a Shannon index of 2.04 and 1.99 for Kraken and MG-RAST, respectively (Table 7). In contrast, the Simpson index was 0.75 and 0.69 for MG-RAST and Kraken, respectively. However, these different values between MG-RAST and Kraken were not significant, suggesting that there is no difference between both algorithms for alpha diversity classification. The observed diversity metrics indicate that the greenhouse water drain is not a diverse niche.

**Table 8:** Taxonomic composition

Phylum	Greenhouse MG-RAST	Greenhouse Kraken
*Acidobacteria*	0.0015405	0.0012883
*Actinobacteria*	0.0267721	0.0316892
*Aquificae*	0.0003382	0.0000208
*Armatimonadetes*	NA	0.0000139
*Bacteroidetes*	0.0208696	0.0049244
*Chlamydiae*	0.0002807	0.0000231
*Chlorobi*	0.0012536	0.0005910
*Chloroflexi*	0.0030123	0.0010620
*Chrysiogenetes*	0.0003128	0.0001454
*Crenarchaeota*	NA	0.0001524
*Cyanobacteria*	0.0047115	0.0015376
*Deferribacteres*	0.0002573	0.0000185
*Deinococcus-Thermus*	0.0024569	0.0021171
*Dictyoglomi*	0.0000570	NA
*Elusimicrobia*	0.0000478	NA
*Euryarchaeota*	NA	0.0005772
*Fibrobacteres*	0.0000351	0.0000277
*Firmicutes*	0.0107219	0.0047444
*Fusobacteria*	0.0002528	0.0000300
*Gemmatimonadetes*	0.0003763	0.0001316
*Ignavibacteriae*	NA	0.0000162
*Lentisphaerae*	0.0001322	NA
*Nitrospirae*	0.0001816	0.0001247
*Planctomycetes*	0.0019784	0.0006049
*Proteobacteria*	0.9125950	0.9467869
*Spirochaetes*	0.0007344	0.0008519
*Synergistetes*	0.0005554	0.0000139
*Tenericutes*	0.0000585	0.0001893
*Thermodesulfobacteria*	NA	0.0000231
*Thermotogae*	0.0004699	0.0001732
*Verrucomicrobia*	0.0099982	0.0021217

Relative abundances at phylum level using MG-RAST and Kraken, Relative abundances were determinate using the  normalized number of reads of each order divided into the total number of reads

### Functional diversity

Next, to know the encoded functions directly from the reads we classified them using MG-RAST and HUMAnN2. According to MG-RAST, we observed that most abundant functions were related to vitamins, protein biosynthesis and central carbohydrate metabolism (Table 9). In contrast, using HUMAnN2 the de Novo nucleotide biosynthesis, vitamins, and prosthetic groups were the most abundant functions (Table 10).

**Table 9:** Functional diversity

Level 2 category	Relative Abundance
Vitamins	0.071243175
Protein biosynthesis	0.055205552
Central carbohydrate metabolism	0.052705254
ABC transporters	0.046491929
Lipids	0.043826646
Disease and Defense	0.037793615
Prophages	0.037587808
Branched-chain amino acids	0.033340704
Arginine	0.032626333
Lysine	0.031525862

Top ten of the most abundant functions annotated by MG-RAST against SEED database. Relative abundances were determined normalizing by the total number of reads

**Table 10:** Functional diversity (UniRef90)

Pathway	Relative abundance
Gondoate biosynthesis	0.071311068
L-isoleucine biosynthesis I	0.058340078
Adenosine ribonucleotides de novo biosynthesis	0.05276
Superpathway of guanosine nucleotides de novo biosynthesis I	0.039785498
L-valine biosynthesis	0.037358283
Guanosine ribonucleotides de novo biosynthesis	0.029089431
Superpathway of pyrimidine deoxyribonucleotides de novo biosynthesis	0.027701595
5-aminoimidazole ribonucleotide biosynthesis II	0.026628851
Superpathway of L-threonine biosynthesis	0.026026654
Mycolate biosynthesis	0.02416827

Top ten of most abundant function annotated by HUMAnN2 against UniRef90 database. Relative abundances were determined normalizing by the total number of reads

### Additional results

**Fig. 2:**
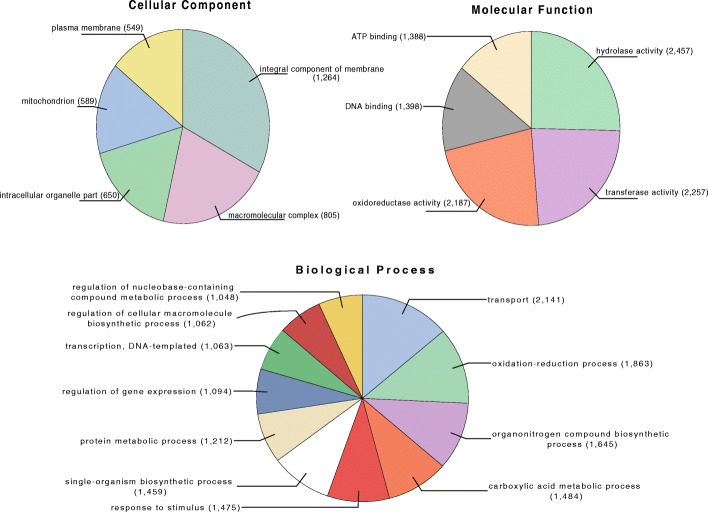
Gene Ontology (GO) terms distribution. The pie graphs show the number of genes annotated for cellular component, biological process, and molecular function categories

**Fig. 3:**
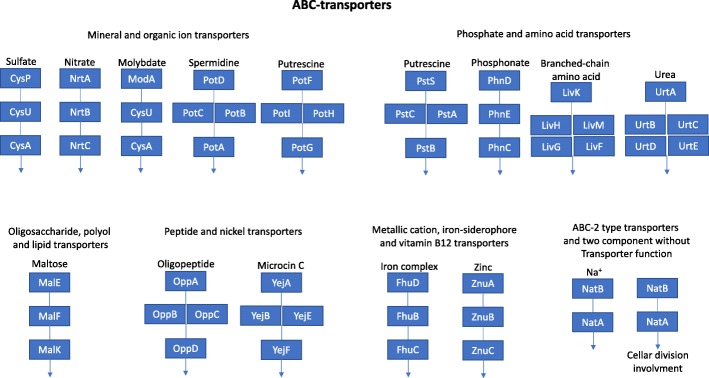
KEGG pathways associated with ABC-transporters. KEGG pathways of the ABC-transporters with all genes present in the metagenome

We assembled the metagenome to get more insight into the protein functional composition of the water sample. In this regard, we used the contigs to predict 25,735 proteins from which only 35.8% had significant blastp match (E-value 10^− 5^) against the NR RefSeq proteins database. The 64.2% of unknown proteins could represent novel proteins. A total of 14,328 (55.6%) proteins were classified using Gene Ontology (GOs) using Blast2GO and Interpro. We found that the term “transport” in the Biological process category was the most abundant function encoded in the greenhouse metagenome (Fig. 2). To get insights on this observation, we found that of the 2141 transporters annotated by Interpro, the 17.23% (369 proteins) are ABC-type transporters. Interestingly, has been reported that *Rhizobiales* has enriched the ABC transporter genes in their genomes (Fig. 2) [31, 32]. Furthermore, we use KAAS-KEGG [33] to identify the pathways containing ABC-transporters. After that, we only found six complete pathways in the greenhouse metagenome such as the vitamin B12 transporter (Fig. 3). Interestingly, the ABC-transporters were also present when we used only the reads for functional analysis (Table 9 and Table 10). Additionally, the hydrolase, transferase, and oxidoreductase were the most abundant GO molecular functions (Fig. 2). This result was in agreement with reports in which rhizobial bacteria associated with the nodules and seed of plants has many genes for these molecular functions in their genomes [34].

Finally, we searched for Antibiotic Resistance Genes (ARGs) in the metagenome, using the ARDB database [35]. We found a total of 31 ARGs and the most abundant genes were for resistance to spectinomycin (16%), tetracycline (12%) and aminoglycosides (9%) (Table 11). These findings are consistent with previous studies in chicken and vegetable greenhouse soil samples [36]. Although ARG genes are common in most ecological niches more investigation is needed to explore the role that these genes could play in microbial dynamics of human-constructed niches such as the greenhouse. Furthermore, these ARGs genes could be used for further analysis in phylogenomics to aim their evolutionary history and trace the adquision of these genes [37].

**Table 11:** Antibiotics resistance genes

Type	Number of genes	Resistance
vatb, aad9ib, aph6ic	5	Spectinomycin
tetpb, tetm, tetx	4	Tetracycline
emre	3	Aminoglycoside
aac3ia	3	Astromicin, Gentamicin, Sisomicin
baca	3	Bacitracin
ceob, catb1	2	Chloramphenicol
cara, tlrc	2	Lincosamide, Macrolide, Streptogramin_b
acrb	1	Acriflavin, Aminoglycoside, beta_lactam, Glycylcycline
bl2d_oxa2	1	Cloxacillin, Penicillin
mexd	1	Erythromycin, Fluoroquinolone, Glycylcycline, Roxithromycin
fosa	1	Fosfomycin
ksga	1	Kasugamycin
macb	1	Macrolide
pbp1b	1	Penicillin
arna	1	Polymyxin
dfra26	1	Trimethoprim

## Conclusions

The use of metagenomic approaches to characterize new environments such as a research-greenhouse has the potential to unveil novel bacterial dynamics, enzyme functions, and metabolic pathways. To the best of our knowledge, this is the first report of the bacterial and functional contribution of the water from a greenhouse drain. We consider it exemplifies how the utilization of a metagenomic approach provides a more comprehensive view regarding the structure and functional composition of a bacterial community. Our results indicated that soil *Rhizobiales* bacteria and their genome functions mainly dominate the greenhouse water drain. This study aimed to identify the bacteria and functional gene composition of a greenhouse water drain sample and also represent a genomic resource to search novel proteins from a previously unexplored niche. Interestingly, we found over 400 proteins containing unintegrated signatures, which are highly conserved domains with unknown function according to Interpro, representing potential novel enzymes. All the metagenome proteins and their annotations are available to the scientific community via http://microbiomics.ibt.unam.mx/tools/metagreenhouse/.

## Abbreviations

ARDB: Antibiotic resistance genes database
ARGs: Antibiotic resistance genes
GO: Gene Ontology
IBt: Institute of Biotechnology
NA: Not available
